# Emergence of Self-Injurious Behaviour in a Student With Autism Following Peer Exposure: A Case Study

**DOI:** 10.1192/bjo.2026.11775

**Published:** 2026-06-30

**Authors:** Nymisha Dangeti, Sherif Abdalla

**Affiliations:** Leicestershire Partnership NHS Trust, Leicester, United Kingdom

## Abstract

**Aims:**

People with autism often exhibit heightened sensitivity to environmental stimuli, including social and emotional cues. Exposure to maladaptive behaviours in peers may act as a powerful trigger, influencing emotional regulation and behavioural responses. This case highlights the impact of environmental modelling on self-harm behaviours in a student with autism.

**Methods:**

We present the case of a student with autism, who developed self-injurious behaviours following exposure to a peer engaging in self-harm at college. The patient demonstrated increased emotional distress, repetitive rumination on the observed behaviour, and subsequent imitation of the act. No acute psychotic symptoms or major mood disorder were identified. The onset of self-harm appeared temporally linked to the environmental exposure rather than internal psychopathology.

**Results:**

This case illustrates the vulnerability of autistic people to social modelling and environmental influences. Heightened empathic sensitivity, difficulties with emotional regulation, and concrete thinking styles may contribute to the internalization and replication of observed behaviours. Early recognition of environmental triggers, particularly within college settings, is crucial. Interventions focused on environmental modification, psychoeducation for caregivers and educators, and autism-adapted emotional regulation strategies were associated with a reduction in self-harm behaviours. This case emphasizes the importance of multidisciplinary collaboration and proactive monitoring of peer-related stressors in autistic populations.

**Conclusion:**

This case underscores the profound environmental sensitivity of people with autism and the potential for peer behaviours to precipitate self-harm through social modelling. Clinicians, caregivers, and educators should remain vigilant to environmental risk factors and prioritize early intervention, college-based collaboration, and psychoeducation. Increased awareness of these vulnerabilities may help prevent self-injurious behaviours and promote safer, more supportive learning environments for students with autism.

